# Human Rhinoviruses in Adult Patients in a Tertiary Care Hospital in Germany: Molecular Epidemiology and Clinical Significance

**DOI:** 10.3390/v13102027

**Published:** 2021-10-08

**Authors:** Philipp Golke, Mario Hönemann, Sandra Bergs, Uwe Gerd Liebert

**Affiliations:** Virology Department, Institute of Medical Microbiology and Virology, Leipzig University, Johannisallee 30, 04103 Leipzig, Germany; Philipp.Golke@medizin.uni-leipzig.de (P.G.); Sandra.Bergs@medizin.uni-leipzig.de (S.B.); Liebert@medizin.uni-leipzig.de (U.G.L.)

**Keywords:** rhinovirus, molecular epidemiology, respiratory infections, respiratory viruses, adult patients

## Abstract

Rhinoviruses (RVs) constitute a substantial public health burden. To evaluate their abundance and genetic diversity in adult patients, RV RNA in respiratory samples was assessed using real-time RT-PCR and the partial nucleic acid sequencing of viral genomes. Additionally, clinical data were retrieved from patient charts to determine the clinical significance of adult RV infections. In total, the respiratory specimens of 284 adult patients (18–90 years), collected from 2013 to 2017, were analyzed. Infections occurred throughout the entire year, with peaks occurring in fall and winter, and showed a remarkably high intra- and interseasonal diversity of RV genotypes. RV species were detected in the following ratios: 60.9% RV-A 173, 12.7% RV-B, and 26.4% RV-C. No correlations between RV species and underlying comorbidities such as asthma (*p* = 0.167), COPD (*p* = 0.312) or immunosuppression (*p* = 0.824) were found. However, 21.1% of the patients had co-infections with other pathogens, which were associated with a longer hospital stay (*p* = 0.024), LRTI (*p* < 0.001), and pneumonia (*p* = 0.01). Taken together, this study shows a pronounced genetic diversity of RV in adults and underlines the important role of co-infections. No correlation of specific RV species with a particular clinical presentation could be deduced.

## 1. Introduction

Rhinoviruses (RV) are highly prevalent and can cause both upper (URTI) and lower respiratory tract infections (LRTI). The clinical spectrum includes common colds [1], otitis media, and sinusitis [2,3,4,5], as well as more severe conditions such as bronchiolitis or pneumonia [2,6]. Moreover, RVs have been associated with the development and exacerbation of asthma and they are the main viral cause of exacerbations of chronic obstructive pulmonary disease (COPD) [7,8,9,10].

Rhinoviruses (RV) belong to the genus *Enterovirus* (EV) within the family of *Picornaviridae*. To date, at least 169 different RV genotypes are known, which can be subdivided into three species: RV-A, RV-B, and RV-C [11]. While RV-A and RV-B have been known since the 1950s, RV-C was first identified in 2006, as it cannot be propagated in standard cell cultures [2,3,12].

RVs are non-enveloped RNA viruses; their positive-sense, single-stranded genome that is approximately 7200 nucleotides in size encodes for viral structural (VP) and non-structural proteins (NSP) [11]. Four proteins, named *VP1*, *VP2*, *VP3*, and *VP4*, form the viral capsid that encases the genome and interacts with the cell surface receptors.

Unlike human enteroviruses or other members of the *Picornaviridae* family, such as parechoviruses, there is a lack of capsid recombination that is characteristic of RV [13]. Thus, the genomic regions of *VP1* and *VP4/VP2* were both proposed for genotypic classification. While the *VP4/VP2* regions allow the genotypic analysis of all three RV species with the same protocol, the *VP1* region offers the highest precision and represents the cornerstone for the assignment of newly discovered genotypes [14].

Though they represent an important public health burden, RVs’ epidemiology and significance remain insufficiently studied. To evaluate the abundance of RVs and the local genetic diversity of RVs in adult patients, the molecular epidemiology of RV was assessed and analyzed between 2013 and 2017 at a tertiary care hospital in Germany. Additionally, the clinical significance of the RV cases and the species-specific clinical significance were analyzed based on the clinical data of the same patient group.

## 2. Materials and Methods

### 2.1. Sample Collection and Clinical Data

From 2013 to 2017, 11,650 respiratory samples from 5400 adult in- and outpatients (>18 years) were collected and tested for viral respiratory infections. Samples included nasal and/or naso-oropharyngeal swabs (16.0%, *n* = 1861), sputum (0.6%, *n* = 74), throat rinsing fluid (65.2%, *n* = 7601), tracheal secretions (3.5%, *n* = 411), and broncho-alveolar lavage fluids (14.6%, *n* = 1703). Testing was initiated at the discretion of the attending physician.

To avoid a bias caused by follow-up samples, re-testing within six weeks after the initial detection was defined as a single case. Data relating to underlying medical conditions and clinical parameters on the day of RV detection were retrieved retrospectively from patient charts. The classification of URTI and LRTI was carried out according to the International Statistical Classification of Diseases and Related Health Problems (ICD-10-WHO) and the diagnoses and information listed in the patients’ records. For LRTI, an individual analysis for pneumonia was presented. Bacterial and fungal pathogens were considered as co-infections if a cultural detection from respiratory samples or blood was documented. Viral co-infections were assessed by a multiplex test for respiratory viruses (see below) or a commercially available CMV assay (Abbott Realtime CMV, Abbott, Chicago, IL, USA). RV seasons were defined as starting on 1 October and ending on 30 September of the following year.

The Leipzig University Ethics committee approved the study design (no. Az 301/16-ek).

### 2.2. Nucleic Acid (NA) Extraction and RV Detection

Total NA was extracted from 200 µL of the respiratory samples using the DNA and Viral NA Small Volume Kit on a MagNA Pure 96 instrument (both, Roche, Mannheim, Germany) according to the manufacturer’s instructions. Nucleic acids were stored in aliquots at −80 °C until further use.

The presence of genomes of common respiratory viruses, including influenza viruses A and B, respiratory syncytial viruses A and B, parainfluenza viruses 1 to 4, human coronaviruses (including 229E, NL63, OC43, and HKU1), human metapneumoviruses, adenoviruses, human bocaviruses, rhinoviruses, and enteroviruses, was assessed using a multiplex panel assay (NxTAG RPP, Luminex corporation, Austin, TX, USA) according to the manufacturer’s instructions. Samples that reacted to the combined enterovirus/rhinovirus target of the assay were further analyzed to determine the presence of rhinovirus-specific RNA. In brief, one-step real-time RT-PCR was performed using the QuantiFast^®^ Multiplex kit (Qiagen, Hilden, Germany) based on a previously proposed protocol [15] and a modified forward primer, which provides increased binding strength due to the incorporation of locked nucleic acids (5′-CY+AGCCTGCGTGGC-3′).

### 2.3. Viral Genotyping and Phylogenetic Analysis

Partial viral *VP1* and *VP4/VP2* genes were amplified for viral genotyping [16,17] using the BigDye Terminator Sequencing Kit v1.1 and an ABI 3500 Genetic Analyzer (both Applied Biosystems, Foster City, CA, USA). The amplicon sizes depended on the RV genotype and were about 340 bp for *VP1* and 420 bp for *VP4/VP2*. If multiple RV-RNA-positive samples of the same patient were available, the first one was used for genotyping. The obtained sequences were submitted to GenBank (accession numbers MZ514934 to MZ515487).

Separate phylogenetic trees for *VP1* and *VP4/VP2* were constructed at the nucleotide level using the MEGA software version 6 based on the maximum likelihood method. Bootstrap analysis was performed with 1000 replicates [18]. The phylogenetic trees included reference sequences proposed by the Picornavirus Study Group [11].

### 2.4. Statistical Analysis

Statistical analyses was performed using IBM SPSS Statistics for Windows, Version 24.0 (Armonk, NY, USA: IBM Corp.). Continuous values were expressed as means or medians (range), and categorical data as frequencies (percentages). A Student’s *t*-test or an ANOVA was performed to compare normally distributed continuous variables. Either a Chi-square or Fisher’s exact test was performed for categorical variables. All tests were two-tailed. A *p*-level of <0.05 was considered significant. 

## 3. Results

### 3.1. Species Distribution and Seasonality

**Figure 1:**
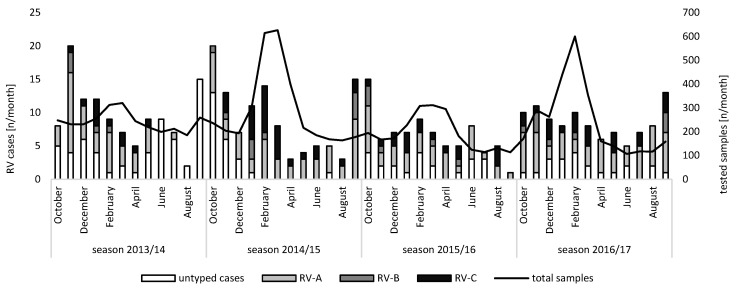
Monthly total numbers of tested samples and rhinovirus cases (*n* = 410) stratified by species A, B, and C, as well as untypable rhinoviruses. Note the two different y-axes: the left axis shows the absolute numbers of detected Rhinovirus A, Rhinovirus B, and Rhinovirus C cases, as well as the absolute numbers of untyped cases, while the right axis shows the absolute number of tested samples.

In total, the presence of RV RNA was confirmed in 506 samples (4.3%) by RT-qPCR. Positivity rates ranged from 4.6% (133/2915) in the season of 2013/2014 to 4.3% (148/3484) in the season of 2014/2015, while they were 4.3% (101/2339) in the season of 2015/2016 and (124/2912) in the season of 2016/2017. Altogether, 410 (3.5%) individual cases were identified. RV infections occurred throughout the whole year without a strict seasonality. However, an increased prevalence was observed between October and November for the season of 2013/2014 to the season of 2015/2016, but not for the season of 2016/2017 (Figure 1).

Partial gene amplification was successful for 284 (69.3%) cases in the *VP4/VP2* region and for 270 (65.9%) cases in the *VP1* region. Due to the insufficient quantities of RNA, genotypic analysis could not be performed in 59 cases (14.4%). In addition, the genotyping approach was unsuccessful for both genetic regions in 67 cases (16.3%). Cases with a successful genotypic analysis were analyzed further. Within the genotyped subset, respiratory specimens included 19 nasal and/or oropharyngeal swabs (6.7%), 4 sputa (1.4%), 193 throat rinsing fluids (68.0%), 12 tracheal secretions (4.2%), and 56 broncho-alveolar lavage fluids (19.7%). Of these, 60 samples (21.1%) were collected in 2013/2014, 86 (30.3%) in 2014/2015, 57 (20.0%) in 2015/2016, and 81 (28.5%) in 2016/2017 (Table 1). Overall, RV-A predominated with 173 detections (60.9%), followed by RV-C with 75 (26.4%) and RV-B with 36 (12.7%) detections. While RV-A and RV-C could be found throughout the entire year, RV-B was not detected from June to August in any of the seasons.

**Figure 2:**
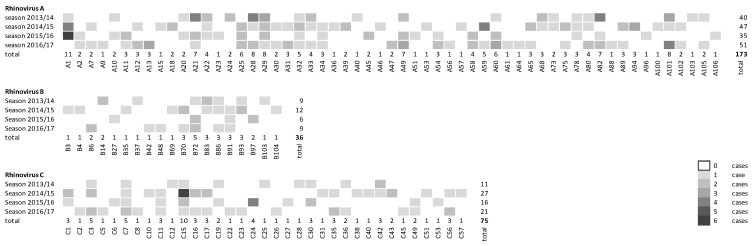
Heat map of the total numbers of RV genotypes (*n* = 284) detected during the study period stratified by season.

Overall, almost two thirds of the established RV genotypes (111/169) were detected in the present case cohort. Of these, four genotypes were shown in all four seasons—RV-A29, RV-A32, RV-C7, and RV-C15—and another 16 could be detected in three seasons (Figure 2).

Regarding the whole study period, the most frequent genotypes of each species were: RV-A1 (3.9%, *n* = 11/284), RV-C15 (3.5%, *n* = 10/284), and RV-B72 (1.8%, *n* = 5/284). No seasonal pattern was shown for the occurrence of specific genotypes either within or between the studied seasons. In the subset of viruses with both *VP1* and *VP4/VP2* genes (*n* = 270), the genotyping results of the two genetic regions matched in all cases. For the 14 samples for which *VP1* sequencing was unsuccessful, the corresponding *VP4/VP2* genotypes were RV-A28, RV-A45, RV-A60, RV-B69, RV-C7, RV-C15, RV-C16, RV-C28, RV-C42, RV-C43, and RV-C56.

### 3.2. Study Population and Clinical Features

The mean age of the study population was 54.8 years, which did not differ significantly between RV species (*p* = 0.193) or between seasons (*p* = 0.957). The gender was female for 37.7% and male for 62.3% of all patients. Again, no difference was noted between seasons (*p* = 0.103) or between RV species (*p* = 0.548) (Table 2).

The RV species-specific patient characteristics and clinical parameters are presented in Table 2. The RV distribution was not statistically different in patients with chronic lung diseases such as asthma (*p* = 0.167) or COPD (*p* = 0.312). The highest frequency of patients with an underlying cardiovascular disease was found for RV-B (*p* = 0.006), representing 69.4% (25/36) of all RV-B cases. Upper and lower respiratory tract infections were noted in 14.7% and 24.6% of all patients, respectively, and did not differ between the RV species detected.

Furthermore, no significant difference in terms of length of stay (*p* = 0.418), ICU stay (*p* = 0.515), or need for ventilation (*p* = 0.648) was observed between the RV species.

### 3.3. Co-Infections

In total, 60 (21.1%) of co-infections were identified, of which 43 (71.7%) were bacterial, 9 (15.0%) were viral, and 3 (5.0%) were fungal. In five cases (8.3%), co-infections with at least two different pathogen types were observed. The most common bacterial pathogens were *Staphylococcus aureus* (*n* = 8), *Pseudomonas aeruginosa* (*n* = 7), *Haemophilus influenzae* (*n* = 6), *Klebsiella pneumoniae* (*n* = 5), and *Escherichia coli* (*n* = 4). Regarding viral and fungal co-pathogens, respiratory syncytial virus (*n* = 3), CMV (*n* = 3), and *Aspergillus spp*. (*n* = 6) were found most frequently (Table 3). No difference was observed for the number of co-infections detected regarding the season (*p* = 0.196) or the gender of the patient (*p* = 0.167). However, LRTI (*p* < 0.001) and pneumonia (*p* = 0.01) were seen more frequently in patients with a co-infection compared to RV mono-infections. Additionally, the need for ventilation and an ICU stay was significantly increased among co-infected patients (*p* = 0.002 and *p* = 0.003, respectively, Table 4).

## 4. Discussion

Rhinoviruses are highly prevalent and constitute a significant public health burden. Therefore, the presented study aimed to investigate the epidemiology and clinical spectrum of rhinovirus infections in adult patients in a German university medical center over four consecutive seasons.

It remains unknown whether a global circulation pattern, comparable to that of influenza viruses, exists for rhinoviruses [19]. However, the detections of identical genotypes all over the globe point to a rapid spread of rhinoviruses and a circulation without geographic limitations [14]. It is remarkable that despite the limited sample set and the local sampling approach used, the majority of the currently proposed RV genotypes were found. The high rhinovirus diversity was evidenced by the different seasons displaying rapidly changing genotype compositions, with the most common genotype RV-A1 representing only 3.9% (11/284) of all detected rhinovirus cases. Only five genotypes, RV-A1, RV-A28 RV-A29, RV-A101, and RV-C15, were found more than seven times (>2.5%) during the study period, making these the most prevalent genotypes. A ubiquitous year-round circulation of a multitude of different RV genotypes has been reported before [20,21,22]. However, the predominance of a single RV genotype in 15.5% [23] and 21.4% [24] of total cases was also described. The observed differences in rhinovirus diversity may be attributed to the divergent study set-ups used. Furthermore, factors originating in the patient population itself may contribute to the more frequent detection of certain RVs. These factors might be an undetected outbreak of RVs within a hospital setting or the introduction of an emerging genotype in a designated geographic area. It is tempting to believe that there are immunological differences that render certain populations more susceptible to infections with different RVs. This could be implied by the development of a serotype-specific humoral immune response [25], representing a correlate of (at least) transient immunity. Consequently, pre-existing immunity could lead to a higher rate of infections with heterologous viral strains, as has been shown for other respiratory viruses [26]. The occurrence of locally restricted and time-limited rhinovirus outbreaks of distinct genotypes has been hypothesized previously [27], which furthermore may contribute to the subtle differences noted in seasonal RV prevalence.

In the present study, rhinovirus detections dropped continuously after February throughout the spring months. Similar seasonal circulation patterns showing a higher detection rate in fall and winter and a decline during the summer months have also been described in Amsterdam [20]. Studies from the USA report a second peak in spring instead of winter [2,21,28]. Then again, RV detection was found to be highest in winter and spring in Nanjing [29]. Despite these differences in rhinovirus seasonality and the vast differences in circulating genotypes, we found a remarkable stability in the ratio of RV species detected. The ratio found in the present study is in line with that seen in previous investigations, with values of 47–64% for RV-A, 2–13% for RV-B, and 21–46% for RV-C [20,30,31,32]. An explanation for this finding might be the different number of genotypes existing for each RV species, which decreases from RV-A to RV-C and RV-B. Potentially, a higher reservoir of genotypes may be more successful in compensating for immunological adaptions in the population and may therefore correlate with the overall RV species abundance.

The extraordinarily high diversity of rhinoviruses may also explain the divergent findings regarding susceptible patient populations and clinical severity. It is of note that associations between clinical differences and RV species seem to have been specific to certain study populations and are not consistently reported. Additionally, the shedding of RV RNA may be observed for more than 30 days in immunocompetent individuals [33,34,35] and was reported for up to two years in a patient with cystic fibrosis [36]. It is thus likely that laboratory detections of RV in clinical samples vary according to the stage of the infection. Thus, the use of differing inclusion criteria mat result in noncongruent study populations and impose major challenges regarding data interpretation and comparability.

Confirming the results of a previous study on hospitalized adults [37], the present dataset did not show a RV species-specific association for all but one underlying comorbidity, which was the presence of cardiovascular disease. This difference may be associated with an increased risk of hospitalization due to cardiovascular risk factors at lower ambient temperatures [38,39]. RV-B was not detected between June and August, which may have led to the higher prevalence of this comorbidity in this specific subgroup compared to RV-A and RV-C.

No RV species could be associated with a higher probability of infections in patients with preexisting respiratory conditions such as COPD, asthma, or structural lung diseases, or in patients who have undergone lung transplantation. The COPD prevalence in the present cohort (14.1%) was higher than that seen in the general population of Germany (5.8%) [40], but consistent with previous studies on influenza and parainfluenza virus type 3 (PIV-3)-infected patients at the same hospital [26,41]. A similar prevalence was also noted for asthma and structural lung diseases when compared to these cohorts.

Furthermore, no differences between the RV species distribution and clinical manifestations or outcome parameters were noted. However, RV species-specific differences have been reported before. For instance, a reduced clinical severity was described for outpatients in comparison to inpatients; this was linked to recombination events in the 5′ non-coding region of RV-A and RV-C [30]. Moreover, a higher rate of ICU admission and a higher rate of pneumonia were seen for RV-A infections [32]. RV-A and RV-B were associated with a greater clinical severity than RV-C [42,43]. In contrast, RV-B was reported to be less pathogenic than RV-A and RV-C, which was attributed to its lower replication efficiency and cytokine production [21,44,45]. In a large European study investigating adult patients with LRTI, RV-A was found to be the most prevalent species [22]. However, the symptom scores and durations were similar for all RV species.

Due to the study site being a tertiary care hospital, severe outcome parameters such as hospitalization itself are likely to be overrepresented. In particular, when compared to influenza B virus-infected patients at the same hospital, the overall clinical severity of RV infections in the current study was lower, as illustrated by their lower number of ICU stays (8.8% vs. 24.9%) and need for invasive ventilation (3.9 vs. 13.3) [26].

The presence of co-infections may be a major contributor to the clinical severity of RV infections. The number of cases of pneumonia (*p* = 0.01) and LRTIs (*p* < 0.001) was increased amongst patients with at least one respiratory co-infection. Co-infections were reported in 66% to 78.9% [32,44] of patients in symptomatic and hospitalized cohorts. However, the discrimination of a combined infection and molecular interactions between RVs and other pathogens is challenging. *Staphylococcus aureus* infections could enhance RV replication within airway epithelia cells in vitro; hence, an underlying *Staphylococcus aureus* colonization could increase susceptibility to RV infections in the airway epithelium [46]. Likewise, it was shown that an infection with *Pseudomonas aeruginosa* in primary bronchial epithelia cells could aggravate cells’ inflammatory response to RV infections [47]. However, there may be a bidirectional influence, as RV infections also seem to promote secondary bacterial infections [2,48], which may be associated with the disruption of the epithelial barrier of the respiratory tract [49]. Thus, the compound effect of having multiple infections could thus enhance the rate of illness and its severity. Correspondingly, the need for ventilation (*p* = 0.002) and for an ICU stay (*p* = 0.003) was significantly increased. Surprisingly, both *Streptococcus pneumoniae* and influenza virus represented only a minority of the detected co-infecting pathogens. One reason for this might be the comparatively low relative positivity rate of RV in respiratory samples during the seasonal peak of invasive pneumococcal disease [50] and influenza virus [26] in February and March (Figure 1). Additionally, for *Streptococcus pneumoniae* the use of an antibiotic before the collection of respiratory samples cannot be ruled out and has been hypothesized before [51].

There are several limitations of this study, including the fact that genotyping was not successful for all detected rhinoviruses, which may be a source of bias, although it was also seen in other studies [44]. A possible explanation for this might be the higher sensitivity of the nucleic acid amplification test used for rhinovirus detection, which targets the highly conserved 5’UTR and amplifies a shorter fragment of 204 bp. Additionally, species-specific differences in the sensitivity of the utilized genotyping protocols cannot be ruled out. Nevertheless, the detection of almost two thirds of the currently proposed rhinovirus genotypes in an overall limited number of patients illustrates the highly diverse circulation pattern of this important respiratory pathogen. Due to the retrospective study design, only associations could be shown, without proof of causality. Finally, patient selection favoring severe cases may have occurred due to our use of sampling at a tertiary care hospital. This is underlined by the prevalence of 14.1% being found for COPD, which was higher than the 5.8% that was found in the general population in Germany [40]. Thus, the high percentage found for LRTI needs to be interpreted with caution.

## 5. Conclusions

This study reports on the epidemiology and associated clinical spectrum of RVs in adult patients who were treated at a tertiary care university hospital in Germany. The present report on circulation patterns is consistent with other studies and highlights the complexity of rhinoviruses’ epidemiology and their tremendous genetic diversity. However, larger epidemiologic studies and surveillance programs are needed for the determination of genotype-specific disease associations, as species-specific differences in underlying comorbidities or clinical severity could not be deduced. However, in addition to preexisting medical conditions, the recognition of co-infections is of profound importance for the assessment of RV-associated disease severity. The vast number of distinct genotypes found necessitates the creation of new treatment and vaccination strategies [52] to overcome the high economic burden of RV-associated illness.

## Figures and Tables

**Table 1:** Gender, age, and rhinovirus species distribution during the study period.

		Season 2013/2014	Season 2014/2015	Season 2015/2016	Season 2016/2017	Total	*p*-Value
mean age	[mean ± SD]	55.25 ± 15.54	54.06 ± 14.59	55.44 ± 17.59	54.78 ± 17.35	54.79 ± 16.15	0.957
female	[% (n/total)]	38.3 (23/60)	38.4 (33/86)	49.1 (28/57)	28.4 (23/81)	37.7 (107/284)	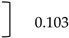
male	[% (n/total)]	61.7 (37/60)	61.6 (53/86)	50.9 (29/57)	71.6 (58/81)	62.3 (177/284)	
RV species							
RV-A	[% (n/total)]	66.7 (40/60)	54.7 (47/86)	61.4 (35/57)	63.0 (51/81)	60.9 (173/284)	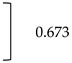
RV-B	[% (n/total)]	15.0 (9/60)	14.0 (12/86)	10.5 (6/57)	11.1 (9/81)	12.7 (36/284)	
RV-C	[% (n/total)]	18.3 (11/60)	31.4 (27/86)	28.1 (16/57)	25.9 (21/81)	26.4 (75/284)	

Analyzed categories are displayed on the column to the left and either given as relative and absolute frequencies (% (*n*/total)) or ranges (median ± SD). (*n*/total) indicates the total amount of cases for the respective season. The brackets indicate parameters that were analyzed in the same contingency table.

**Table 2:** Study population and clinical features of RV cases.

		RV-A 60.9% (173/284)	RV-B 12.7% (36/284)	RV-C 26.4% (75/284)	Total	*p*-Value
**study population**						
female	[% (n/total)]	36.4 (63/173)	33.3 (12/36)	42.7 (32/75)	37.7 (107/284)	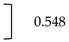
male	[% (n/total)]	63.6 (110/173)	66.7 (24/36)	57.3 (43/75)	62.3 (177/284)	
age[years]	[mean ± SD]	54.4 ± 17.24	51.44 ± 13.7	57.31 ± 14.34	54.79 ± 16.15	0.193
inpatients	[% (n/total)]	64.2 (111/173)	69.4 (25/36)	68.0 (51/75)	65.8 (187/284)	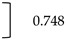
outpatients	[% (n/total)]	35.8 (62/173)	30.6 (11/36)	32.0 (24/75)	34.2 (97/284)	
length of stay [days]	[median(range)]	9.0 (1–130)	9.0 (1–36)	8.0 (0–82)	9 (1–130)	0.364
**comorbidities and risk factors**						
asthma	[% (n/total)]	5.2 (9/173)	8.3 (3/36)	12.0 (9/75)	7.4 (21/284)	0.167
COPD	[% (n/total)]	13.3 (23/173)	22.2 (8/36)	12.0 (9/75)	14.1 (40/284)	0.312
structural lung disease	[% (n/total)]	2.9 (5/173)	5.6 (2/36)	8.2 (6/75)	4.6 (13/284)	0.178
lung transplant	[% (n/total)]	11.6 (20/173)	13.9 (5/36)	12.0 (9/75)	12.0 (34/284)	0.926
chronic kidney failure	[% (n/total)]	21.4 (37/173)	16.7 (6/36)	30.7 (23/75)	23.2 (66/284)	0.172
cardiac insuffiency	[% (n/total)]	6.9 (12/173)	5.6 (2/36)	5.3 (4/75)	6.3 (18/284)	0.937
cardiovascular diseases	[% (n/total)]	41.6 (72/173)	69.4 (25/36)	53.3 (40/75)	48.2 (137/284)	0.006
diabetes	[% (n/total)]	23.7 (41/173)	16.7 (6/36)	17.3 (13/75)	21.1 (60/284)	0.414
malignancy	[% (n/total)]	41.0 (71/173)	30.6 (11/36)	49.3 (37/75)	41.9 (119/284)	0.161
immunosuppression	[% (n/total)]	37.0 (64/173)	41.7 (15/36)	40.0 (30/75)	38.4 (109/284)	0.824
**clinical presentation and features**						
fever	[% (n/total)]	24.4 (19/78)	13.3 (2/15)	22.6 (7/31)	22.6 (28/124)	0.646
URTI	[% (n/total)]	17.6 (26/148)	18.8 (6/32)	15.4 (10/65)	17.1 (42/245)	0.897
LRTI	[% (n/total)]	29.1 (43/148)	31.3 (10/32)	26.2 (17/65)	28.6 (70/245)	0.854
pneumonia	[% (n/total)]	22.3 (33/148)	34.4 (11/32)	20.0 (13/65)	23.3 (57/245)	0.262
co-infection	[% (n/total)]	23.1 (40/173)	13.9 (5/36)	20.0 (15/75)	21.1 (60/284)	0.449
bacterial	[% (n/total)]	16.8 (29/173)	13.9 (5/36)	12.0 (9/75)	15.1 (43/284)	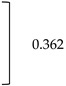
viral	[% (n/total)]	2.3 (4/173)	0.0 (0/36)	6.7 (5/75)	3.2 (9/284)	
fungal	[% (n/total)]	1.7 (3/173)	0.0 (0/36)	0.0 (0/75)	1.1 (3/284)	
combined	[% (n/total)]	2.3 (4/173)	0.0 (0/36)	1.3 (1/75)	1.8 (5/284)	
ICU stay	[% (n/total)]	8.1 (14/173)	13.9 (5/36)	8.0 (6/75)	8.8 (25/284)	0.515
ventilation	[% (n/total)]	9.2 (16/173)	8.3 (3/36)	14.7 (11/75)	10.6 (30/284)	0.398
non-invasive	[% (n/total)]	6.4 (11/173)	5.6 (2/36)	8.0 (6/75)	6.7 (19/284)	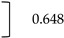
invasive	[% (n/total)]	2.9 (5/173)	2.8 (1/36)	6.7 (5/75)	3.9 (11/284)	

Analyzed categories are displayed on the column to the left and are either given as frequencies (%), medians, and ranges (median (range)) or as means and standard deviations (mean ± SD). (*n*/total) indicates the respective cases for the total amount of available data. The brackets indicate parameters that were analyzed in the same contingency table. Statistically significant p-values (*p* < 0.05) are in bold. COPD, chronic obstructive pulmonary disease; URTI, upper respiratory tract infection; LRTI, lower respiratory tract infection; ICU, intensive care unit.

**Table 3:** Distribution of co-infecting pathogens.

Bacteria	*n*	Viruses	*n*	Fungi	*n*
*Staphylococcus aureus*	8	RSV	3	*Aspergillus spp.*	6
*Pseudomonas aeruginosa*	7	CMV	3	*Pneumocystis jirovecii*	1
*Haemophilus influencae*	6	Coronavirus 229 E	1		
*Klebsiella Pneumoniae*	5	Coronavirus OC 43	1		
*Escherichia coli*	4	Enterovirus B (Echovirus 18)	1		
*Haemophilus parainfluenzae*	3	Influenza A Virus H3N2	1		
*Serratia marcescens*	3	Parainfluenza Typ 4	1		
*Streptococcus pneumoniae*	3				
*Acinetobacter baumanii*	2				
*Burkholderia cenocepacia*	2				
*Chlamydophila pneumoniae*	2				
*Enterococcus faecium*	2				
*Haemophylus parahaemolyticus*	2				
*Proteus mirabilis*	2				
*Citrobacter freundii*	1				
*Enterococcus faecalis*	1				
*Klebsiella oxytoca*	1				
*Mycobacterium kansasii*	1				
*Mykobakterium tuberkulosis*	1				
*Raoutella planticola*	1				
*staphylococcus hämolyticus*	1				
*Streptococcus pyogenes*	1				
*Streptococcus mitis*	1				

Pathogens detected by type, with n showing their frequencies of detection.

**Table 4:** Comparison of samples with and without co-infections.

		RV Only	RV + Co-Infection	Total	*p*-Value
**total**	[% (*n*/total)]	78.9 (224/284)	21.1 (60/284)	284	
**season**					
season 2013/2014	[% (*n*/total)]	23.2 (52/224)	13.3 (8/60)	21.1 (60/284)	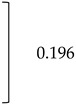
season 2014/2015	[% (*n*/total)]	31.3 (70/224)	26.7 (16/60)	30.3 (86/284)	
season 2015/2016	[% (*n*/total)]	19.2 (43/224)	23.3 (14/60)	20.1 (57/284)	
season 2016/2017	[% (*n*/total)]	26.3 (59/224)	36.7 (22/60)	28.5 (81/284)	
**population**					
female	[% (*n*/total)]	39.7 (89/224)	30.0 (18/60)	37.7 (107/284)	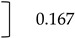
male	[% (*n*/total)]	60.3 (135/224)	70.0 (42/60)	62.3 (177/284)	
**sample origin**					
upper airways	[% (*n*/total)]	79.0 (177/224)	66.7 (40/60)	76.4 (217/284)	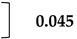
lower airways	[% (*n*/total)]	22.3 (47/224)	27.4 (20/60)	23.6 (67/284)	
**clinical presentation**					
URTI	[% (*n*/total)]	20.4 (38/186)	6.8 (4/59)	14.8 (42/284)	**0.015**
LRTI	[% (*n*/total)]	22.6 (42/186)	47.5 (28/59)	24.6 (70/284)	**<0.001**
pneumonia	[% (*n*/total)]	19.4 (36/186)	35.6 (21/59)	20.0 (57/284)	**0.01**
lung transplant	[% (*n*/total)]	11.2 (25/224)	15.0 (9/60)	12.0 (34/284)	0.416
ICU stay	[% (*n*/total)]	6.3 (14/224)	18.3 (11/60)	8.8 (25/284)	**0.003**
ventilation	[% (*n*/total)]	7.6 (17/224)	21.7 (13/60)	10.6 (30/284)	**0.002**
non-invasive	[% (*n*/total)]	5.8 (13/224)	10.0 (6/60)	6.7 (19/284)	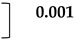
invasive	[% (*n*/total)]	1.8 (4/224)	11.7 (7/60)	3.9 (11/284)	
length of stay [days] *	[median (range)]	7.0 (1–87)	11.0 (1–82)	8.0 (1–87)	**0.024**

Analyzed categories are displayed on the column to the left and either given as frequencies (%) or medians and ranges (median (range)). (*n*/total) indicates the respective cases for the total amount of available data. A comparison between RV cases with (RV + coinfection) and without (RV only) a documented coinfection with a viral, bacterial, or fungal pathogen was performed. * Only inpatients were included. The brackets indicate parameters that were analyzed in the same contingency table. Statistically significant p-values (*p* < 0.05) are in bold.

## Data Availability

Identified sequences were submitted to GenBank (accession no. MZ514934–MZ515487).
